# Joint relationship between renal function and proteinuria on mortality of patients with type 2 diabetes: The Taichung Diabetes Study

**DOI:** 10.1186/1475-2840-11-131

**Published:** 2012-10-19

**Authors:** Cheng-Chieh Lin, Ching-Chu Chen, Pei-Tseng Kung, Chia-Ing Li, Sing-Yu Yang, Chiu-Shong Liu, Wen-Yuan Lin, Cheng-Chun Lee, Tsai-Chung Li, Sharon LR Kardia

**Affiliations:** 1 Department of Family Medicine, China Medical University Hospital, Taichung, Taiwan; 2 School of Medicine, College of Medicine, China Medical University, Taichung, Taiwan; 3 Department of Medical Research, China Medical University Hospital, Taichung, Taiwan; 4 Division of Endocrinology and Metabolism, Department of Medicine, China Medical University Hospital, Taichung, Taiwan; 5 Department of Healthcare Administration, College of Health Science, Asia University, Taichung, Taiwan; 6 Graduate Institute of Biostatistics, College of Public Health, China Medical University, 91 Hsueh-Shih Road, Taichung 40421, Taiwan; 7 Department of Neurology, China Medical University Hospital, Taichung, Taiwan; 8 Graduate Institute of Clinical Medical Science, College of Medicine, China Medical University, Taichung, Taiwan; 9 Department of Epidemiology, University of Michigan, Ann Arbor, MI, USA

**Keywords:** Renal function, Mortality, Type 2 diabetes

## Abstract

**Background:**

Estimated glomerular filtration rate (eGFR) is a powerful predictor of mortality in diabetic patients with limited proteinuria data. In this study, we tested whether concomitant proteinuria increases the risk of mortality among patients with type 2 diabetes.

**Methods:**

Participants included 6523 patients > 30 years with type 2 diabetes who were enrolled in a management program of a medical center before 2007. Renal function was assessed by eGFR according to the Modification of Diet in Renal Disease Study equation for Chinese. Proteinuria was assessed by urine dipstick.

**Results:**

A total of 573 patients (8.8%) died over a median follow-up time of 4.91 years (ranging from 0.01 year to 6.42 years). The adjusted expanded cardiovascular disease (CVD)-related mortality rates among patients with proteinuria were more than three folds higher for those with an eGFR of 60 mL/min/1.73 m^2^ or less compared with those with an eGFR of 90 mL/min/1.73 m^2^ or greater [hazard ratio, HR, 3.15 (95% confidence interval, CI, 2.0–5.1)]. The magnitude of adjusted HR was smaller in patients without proteinuria [1.98 (95% CI, 1.1–3.7)]. An eGFR of 60 mL/min/1.73 m^2^ to 89 mL/min/1.73 m^2^ significantly affected all-cause mortality and mortality from expanded CVD-related causes only in patients with proteinuria. Similarly, proteinuria affected all outcomes only in patients with an eGFR of <60 mL/min/1.73 m^2^.

**Conclusion:**

The risks of all-cause mortality, as well as expanded and non-expanded mortality from CVD-related causes associated with proteinuria or an eGFR of 90 mL/min/1.73 m^2^ or greater are independently increased. Therefore, the use of proteinuria measurements with eGFR increases the precision of risk stratification for mortality.

## Introduction

Diabetic nephropathy is the most common cause of end-stage renal disease (ESRD) worldwide and is the primary indication for renal replacement therapy in most developed countries [1,2]. ESRD is strongly associated with cardiovascular morbidity and mortality [3], thereby putting a consid0erable burden on healthcare systems worldwide [1-4]. Meanwhile, the incidence and prevalence rates of ESRD have continuously increased in Taiwan since 2000 [4]. According to previous epidemiological studies, the key predictors of chronic kidney disease include age, female sex, diabetes [5], hypertension [5] or hypertension medication [5,6], and hyperlipidemia [7]. Meanwhile, diabetes is rapidly increasing in the Chinese population, especially in Taiwan, mainland China, Hong Kong, and Singapore, accounting for at least one-fifth of the global population [8-11].

Although diabetes is the leading cause of decreased renal function, diabetes itself is an important predictor of cardiovascular morbidity and mortality [12]. Several studies have explored the association between estimated glomerular filtration rate (eGFR) and all-cause mortality and cardiovascular-specific morbidity and mortality [13-31]. However, the studies were conducted involving patients with cardiovascular disease [15-20], patients with impaired kidney function [21,22,32], or a general population [23-28]. Only a few studies have explored the relationship between decreased renal function (expressed as eGFR) and mortality in patients with diabetes [13,14,29-31], and few studies identified the relationship between eGFR and CVD-specific mortality [13,31]. In addition, it has been reported that proteinuria was independently associated with cardiovascular events among patients with type 2 diabetic nephropathy and hypertension when reduced eGFR was considered [33]. And there is a need to explore the association between eGFR and mortality by stratifying proteinuria status in patients with type 2 diabetes.

The current study aims to examine the association between annual eGFR and the all-cause and the expanded, and non-expanded CVD-related mortalities by stratifying the proteinuria status in a large cohort of ethnic Chinese patients with type 2 diabetes, who were followed for more than 4 years at a single medical center.

## Methods

### Study population

Patients in this prospective cohort were selected from all participants of the Diabetes Care Management Program (DCMP) of the China Medical University Hospital (CMUH), Taichung, Taiwan. DCMP is a case-management program established by the Taiwan Bureau of National Health Insurance in 2002 that provides an intervention program aimed at meeting treatment goals recommended by the American Diabetes Association (ADA) by tightly controlling multiple factors. All patients with a clinically confirmed diagnosis of diabetes mellitus per the ADA criteria (International Classification of Diseases, ninth revision, clinical modification, ICD-9-CM; Diagnosis code 250) were invited to participate. This program provides financial incentives for physicians to increase exhaustive follow-up visits, develop an educational program for diet and life style behaviors, and conduct examinations and four laboratory tests annually. In addition, participants were required to complete a standardized, computerized questionnaire administered by a case management nurse to record previous or current disease status, as well as lifestyle behaviors. Patients with type 1 diabetes (ICD-9-CM; Code of 250.x1/x3) and those with an eGFR value of <15 mL/min/1.73 m^2^ were excluded.

Participants who were enrolled in the DCMP-CMUH by the end of August 2007 were identified from an automated registry. All patients who enrolled in the registry from August 2002 to August 2006 and had been continuously enrolled in the program until August 2008 or until death were included. The inclusion criteria was stipulated for patients with type 2 diabetes who can provide at least one year of follow-up for eGFR estimation and one year of follow-up for evaluating the outcome measures. These criteria were met by 6523 patients with type 2 diabetes. The study was approved by the Ethics Review Board of the China Medical University Hospital.

Blood was drawn with minimal trauma from an antecubital vein in the morning after a 12-hour overnight fasting, and was sent for analysis within four hours of collection. Biochemical markers, such as serum creatinine, fasting plasma glucose (FPG), high-density lipoprotein cholesterol (HDL-C), and triglycerides were analyzed with a biochemical autoanalyzer (Beckman Coulter Synchron System, Lx-20, Fullerton, CA, USA) at the Clinical Laboratory Department of CMUH. Serum cholesterol and triglyceride levels were determined by enzymatic colorimetry. HDL-C and low-density lipoprotein cholesterol (LDL-C) levels were measured by direct HDL-C method. Hemoglobin A_1_C (glycosylated hemoglobin) was measured using a boronate-affinity high-performance liquid chromatography (HPLC) assay (reference range, 4.6% to 6.5%). The inter- and intra-assay coefficients of variation (CV) for HbA_1_C were 2.91% for the normal level, 1.79% for the intermediate level, and 1.09% for the high level. Modification of Diet in Renal Diseases (MDRD) Study equation was used to estimate GFR based on serum creatinine levels [34], where estimated GFR (eGFR in mL/min/1.73 m^2^) = 186 × (standardized serum creatinine in mg/dl)^-1.154^ × age^-0.203^ (× 0.742, if female, and ×1.233 if Chinese) [35]. Decreased eGFR was defined as eGFR ≤ 60 mL/min/1.73 m^2^ and > 15 mL/min/1.73 m^2^[34], which correspond to stages 3 and 4. Baseline urine dipstick measurement was used to define proteinuria and was classified as absence (negative urine dipstick reading), presence (urine dipstick reading trace, 1+, or ≥2+).

### Outcome measures

Primary outcome measures were all-cause mortality and mortality due to expanded and non-expanded CVD-related disease [36]. Expanded CVD-related disease was considered because of the high likelihood of classifying death due to CVD in patients with diabetes as death due to diabetes, whereas, in patients with diabetes and kidney disease, as death due to kidney disease. Thus, we derived the composite measures of expanded and non-expanded CVD mortality by grouping all deaths due to CVD, diabetes or kidney disease as expanded CVD mortality and the other causes of deaths as non-expanded CVD mortality, which was first used by Wen et al. [36]. Expanded CVD mortality was defined as deaths due to CVD (ICD-9-CM; Diagnosis codes 390–459), diabetes (ICD-9-CM; Diagnosis code 250), or kidney disease (ICD-9-CM; Diagnosis code 580–589), whichever occurred first. The Taiwan National Death Index, a database that contains records of deaths in the Taiwanese population, was used to identify possible decedents during the follow-up period. Deaths were confirmed by our registry after they had been identified. Linking the unique identification numbers with this computerized file, 596 deaths were identified in the cohort studied by the end of 2008. Deaths were coded according to the ICD-9-CM.

### Statistical analyses

Patients were stratified into three stages of renal function based on annual mean eGFR values according to the Kidney Disease Outcomes Quality Initiative guidelines as follows: stage 1 (eGFR, ≥ 90 mL/min/1.73 m^2^), stage 2 (eGFR, 60 mL/min/1.73 m^2^ to 89 mL/min/1.73 m^2^), stage 3 (eGFR, 30 mL/min/1.73 m^2^ to 59 mL/min/1.73 m^2^), and stage 4 (eGFR, 15 mL/min/1.73 m^2^ to 29 mL/min/1.73 m^2^).

Kaplan-Meier cumulative incidence plots were generated, showing time-to-event for all end points. Time-dependent Cox proportional hazards models were used to evaluate the association between the stages of renal function and mortality by considering the time-varying stage of renal function. Hazard ratios and their 95% confidence intervals (CI) of eGFR and proteinuria were calculated by adjusting for age and multiple variables. Two multivariate models were used. The first multivariate model adjusted for age (continuous), hypertension (yes, no), antihypertensive treatment (yes, no), smoking (yes, no), obesity (yes, no), alcohol consumption (yes, no), exercise (yes, no), duration of diabetes, hyperlipidemia (yes for total cholesterol ≥ 200 mg/dl, or triglycerides ≥ 150 mg/dl, no), type of treatment (oral hypoglycemic drug, insulin injection, both, or both not), and HbA_1_C. In addition to the variables in the first model, the second model adjusted for complications at the baseline (diabetic ketoacidosis, hyperglycemia hyperosmolar non-ketoacidosis, severe hypoglycemia, stroke, myocardial infarction, peripheral neuropathy, intermittent claudication, and neuropathy). The P values for the trends in annual mean eGFR across stages were calculated. All P values were two-tailed, and P < 0.05 was considered statistically significant. All analyses were performed using the statistical package SAS for Windows (Version 9.2, SAS, Cary, NC, USA).

## Results

**Figure 1:**
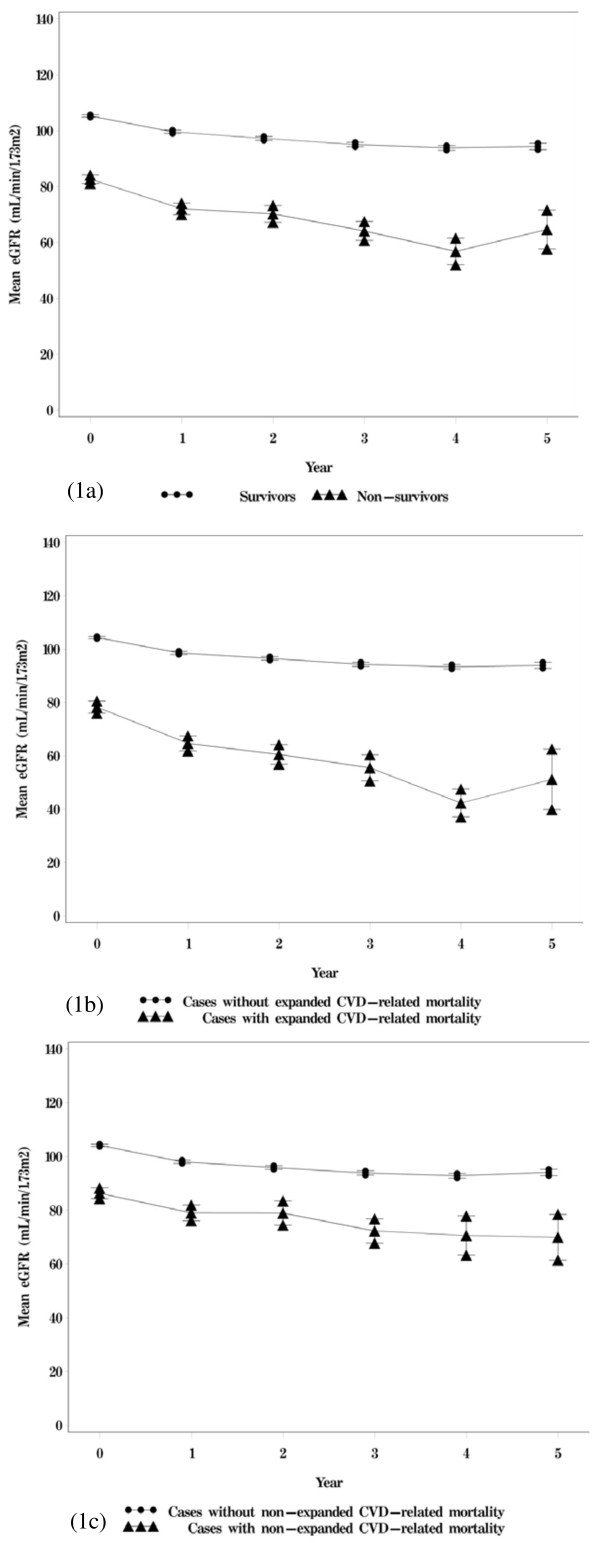
Mean annual eGFR according to (A) survival status, (B) status of expanded CVD-mortality, and (C) status of non-expanded CVD-mortality.

The mean follow-up was 4.31 years. Among the 6523 patients, 573 died during the follow-up period. The crude mortality rate was 20.37 per 1000 person-years (24.78 per 1000 person-years for men and 15.75 per 1000 person-years for women). Cancer was the leading cause of death (n=166, crude rate = 5.90 per 1000 person-years), followed by diabetes (n=132, crude rate = 4.69 per 1000 person-years), and cardiovascular disease (n=105, crude rate = 3.73 per 1000 person-years). These causes accounted for a total of 70.33% of all deaths. Among the patients with type 2 diabetes, 2023, 1355, 927, 791, and 1427 patients provided 1,2, 3, 4, and ≥5 years of annual eGFR measurements, respectively. The correlation coefficients among annual eGFR from years 1 to 5 ranged from 0.78 to 0.94. Figure 1 shows the means and standard errors of eGFR from years 1 to 5 according to the statuses of all-cause mortality and mortality due to expanded and non-expanded CVD-related disease. Individuals with all-cause mortality and mortality due to expanded and non-expanded CVD-related disease were associated with lower levels of eGFR from years 1 to 5.

Baseline eGFR ≥ 90 mL/min/1.73 m^2^ was associated with lower mean age, shorter duration of diabetes medication, lower levels of triglyceride, higher levels of HDL-C, HbA1c and FPG, and higher prevalence of smoking and alcohol drinking statuses, and lower prevalence of exercise, taking oral hypoglycemic agents and insulin, hypertension, hyperlipidemia, hyperglycemia hyperosmolar non-ketoacidosis, severe hypoglycemia, stroke, coronary artery disease, myocardial infarction, peripheral neuropathy, intermittent claudication, and neuropathy (Table 1).

**Table 1:** Comparisons of baseline sociodemographic factors, lifestyle behaviors, diabetes-related variables, drug-related variables, diabetes-related diseases, and blood biochemical indices among patients with different clinical stages of baseline renal function, as determined by mean eGFR

**Variables**	**Baseline mean eGFR N (%)**			**P value**
	**≥90 (N=3686)**	**60~89 (N=1981)**	**15~59 (N=856)**	
***Sociodemographic factors***				
Gender				0.01
Female	1800 (48.83)	898 (45.33)	432 (50.47)	
Male	1886 (51.17)	1083 (54.67)	424 (49.53)	
Age (yrs)†	54.16±10.96	64.19±10.18	67.22±10.09	<0.001
***Lifestyle behaviors***				
Smoking				<0.001
No	2863 (77.67)	1665 (84.05)	724 (84.58)	
Yes	823 (22.33)	316 (15.95)	132 (15.42)	
Alcohol drinking				<0.001
No	3200 (86.81)	1784 (90.06)	810 (94.63)	
Yes	486 (13.19)	197 (9.94)	46 (5.37)	
Exercising				<0.001
No	1650 (44.76)	702 (35.44)	304 (35.51)	
Yes	2036 (55.24)	1279 (64.56)	552 (64.49)	
***Diabetes-related variables***				
Diabetes medical history(yrs)†	5.22±6.06	7.26±7.11	11.23±8.57	<0.001
Type of DM treatment				<0.001
Oral hypoglycemic drug	3045 (82.61)	1706 (86.12)	659 (76.99)	
Inject insulin	69 (1.87)	15 (0.76)	10 (1.17)	
Both	261 (7.08)	159 (8.03)	167 (19.51)	
Both not	311 (8.44)	101 (5.10)	20 (2.34)	
***Drug-related variables***				
Hypertension drug treatment				<0.001
No	2543 (68.99)	953 (48.11)	295 (34.46)	
Yes	1143 (31.01)	1028 (51.89)	561 (65.54)	
***Diabetes-related diseases***				
Obesity				0.18
No	2581 (70.02)	1346 (67.95)	580 (67.76)	
Yes	1105 (29.98)	635 (32.05)	276 (32.24)	
Hypertension				<0.001
No	2517 (68.29)	967 (48.81)	305 (35.63)	
Yes	1169 (31.71)	1014 (51.19)	551 (64.37)	
Hyperlipidemia				<0.001
No	2637 (71.54)	1317 (66.48)	549 (64.14)	
Yes	1049 (28.46)	664 (33.52)	307 (35.86)	
DKA				0.72
No	3644 (98.86)	1963 (99.09)	847 (98.95)	
Yes	42 (1.14)	18 (0.91)	9 (1.05)	
HHNK				<0.001
No	3642 (98.81)	1926 (97.22)	813 (94.98)	
Yes	44 (1.19)	55 (2.78)	43 (5.02)	
Severe Hypoglycemia				<0.001
No	3624 (98.32)	1942 (98.03)	810 (94.63)	
Yes	62 (1.68)	39 (1.97)	46 (5.37)	
Stroke				<0.001
No	3522 (95.55)	1799 (89.80)	722 (84.35)	
Yes	164 (4.45)	202 (10.20)	134 (15.65)	
Coronary artery disease				<0.001
No	3546 (96.20)	1799 (90.81)	726 (84.81)	
Yes	140 (3.80)	182 (9.19)	130 (15.19)	
Myocardial infarction				<0.001
No	3414 (92.62)	1805 (91.12)	748 (87.38)	
Yes	272 (7.38)	176 (8.88)	108 (12.62)	
Peripheral neuropathy				<0.001
No	3201 (86.84)	1660 (83.80)	600 (70.09)	
Yes	485 (13.16)	321 (16.20)	256 (29.91)	
Intermittent claudication				<0.001
No	3641 (98.78)	1946 (98.23)	829 (96.85)	
Yes	45 (1.22)	35 (1.77)	27 (3.15)	
Neuropathy				<0.001
No	3461 (93.90)	1823 (92.02)	696 (81.31)	
Yes	225 (6.10)	158 (7.98)	160 (18.69)	
***Blood biochemical indexes***				
Triglyceride (mg/dl) †	159.98±205.49	166.44±191.09	190.26±216.96	<0.001
High-density lipoprotein (mg/dl) †	41.76±11.31	40.15±10.91	39.30±11.68	<0.001
HbA1c (%)†	8.59±2.01	8.11±1.89	8.26±1.92	<0.001
Fasting plasma glucose (mg/dl) †	169.61±58.40	161.14±59.83	162.37±69.18	<0.001

†:mean±SD; DDK: Diabetic ketoacidosis; HHNK: Hyperglycemia hyperosmolar non-ketoacidosis.

Differences in continuous variables were tested by the student’s *t*-test.

Differences in categorical variables were tested by the Chi-square test.

**Figure 2:**
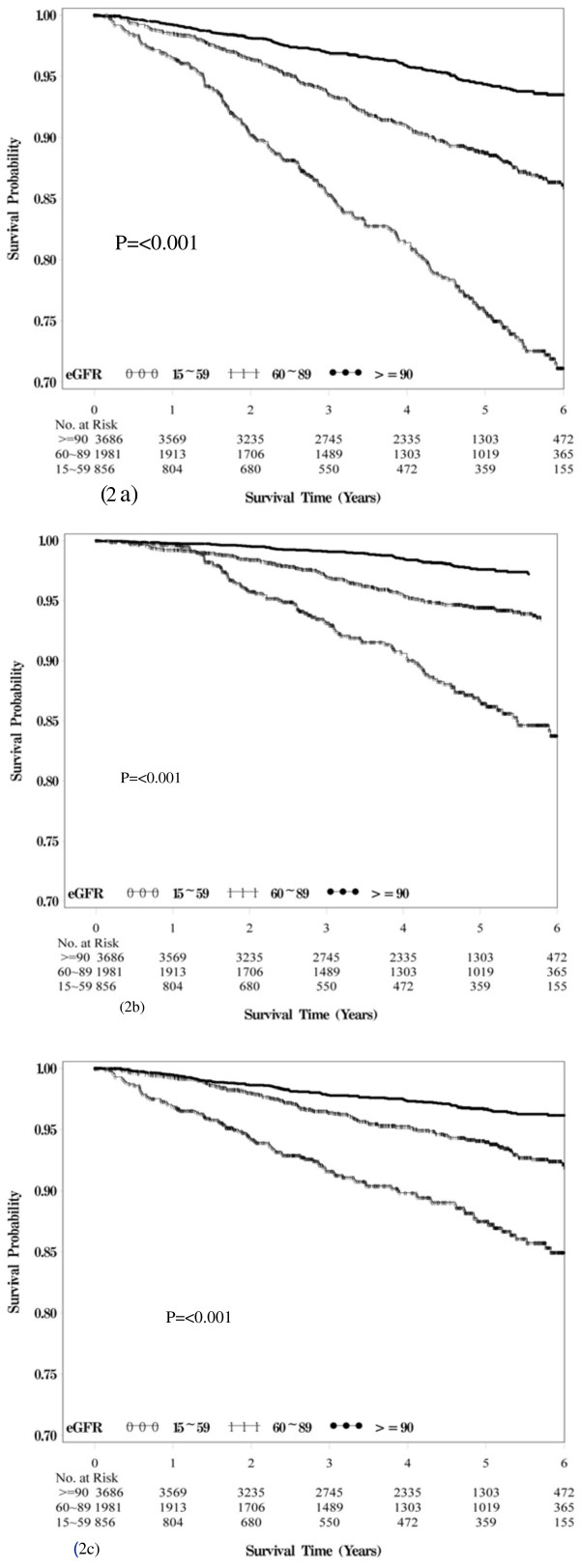
Survival curves of (a) death from all causes, (b) from expanded CVD, and (c) from non-expanded CVD for baseline eGFR of various clinical stages.

Figure 2 presents the Kaplan-Meier survival curves for all-cause and expanded and non-expanded CVD-related mortality within the subgroups defined by the baseline eGFR. Median survival ranged from 4.83 years for patients with eGFR ranging from 15 mL/min/1.73 m^2^ to 59 mL/min/1.73 m^2^ to 4.66 years for patients with eGFR ≥ 90 mL/min/1.73 m^2^. Baseline eGFR was a powerful predictor of all-cause mortality, as well as for both expanded and non-expanded CVD-related mortalities. Patients with eGFR values ranging from 15 mL/min/1.73 m^2^ to 59 mL/min/1.73 m^2^ were at increased risk of death (log-rank p<0.001, Figure 2a), mainly from expanded (log-rank p<0.001, Figure 2b) and non-expanded CVD-related causes (log-rank p<0.001, Figure 2c).

Table 2 shows the hazard ratios (HRs) of mortality resulting from all causes, as well as from expanded and non-expanded CVD-related causes according to the stages of mean annual eGFR and proteinuria. Patients with a mean annual eGFR of 15 mL/min/1.73 m^2^ to 59 mL/min/1.73 m^2^ were at a 2.37-fold greater risk of all-cause mortality (95% CI, 1.88–2.99), a 2.74-fold greater risk of expanded CVD-related mortality (95% CI, 1.92–3.90), and at a 2.13-fold greater risk of non-expanded CVD-related mortality (95% CI, 1.56–2.91) compared with patients with a mean annual eGFR ≥ 90 mL/min/1.73 m^2^after considering age, lifestyle behaviors, medications, glucose control, and complications. The corresponding HRs of proteinuria for all-cause and for both expanded and non-expanded CVD-related mortalities were 1.55 (95% CI, 1.28–1.87), 1.63 (95% CI, 1.22–2.16), and 1.49 (95% CI, 1.16–1.92). Significant linear trends were found across the stages of annual mean eGFR for all-cause mortality, and mortality due to expanded and non-expanded CVD-related causes.

**Table 2:** Hazard ratios (HRs) of all-cause mortality and mortality due to expanded and non-expanded CVD-related causes based on the clinical stage of the time-dependent annual mean eGFR

**Variables**	**All-cause mortality**	**P for trend**	**Expanded CVD-related mortality**	**P for trend**	**Non-expanded CVD-related mortality**	**P for trend**
	**HR (95% CI)**		**HR (95% CI)**		**HR (95% CI)**	
Age-adjusted						
Annual mean eGFR (mL/min/1.73m^2^)		<0.001		<0.001		<0.001
≥90	1.00		1.00		1.00	
60~89	1.23 (0.99-1.53)		1.41 (1.00-2.00)*		1.13 (0.85-1.51)	
15~59	2.37 (1.90-2.97)***		2.90 (2.07-4.10)***		2.04 (1.51-2.75)***	
Proteinuria	1.83 (1.53-2.19)***		1.99 (1.52-2.61)***		1.71 (1.35-2.18)***	
Multivariate-adjusted^1^						
Annual mean eGFR (mL/min/1.73m^2^)		<0.001		<0.001		<0.001
≥90	1.00		1.00		1.00	
60~89	1.33 (1.07-1.66)*		1.55 (1.09-2.19)*		1.21 (0.90-1.62)	
15~59	2.49 (1.98-3.14)***		3.01 (2.12-4.28)***		2.16 (1.59-2.94)***	
Proteinuria	1.62 (1.35-1.95)***		1.75 (1.33-2.31)***		1.53 (1.20-1.95)***	
Multivariate-adjusted^2^						
Annual mean eGFR (mL/min/1.73m^2^)		<0.001		<0.001		<0.001
≥90	1.00		1.00		1.00	
60~89	1.31 (1.05-1.63)*		1.50 (1.06-2.12)*		1.20 (0.90-1.62)	
15~59	2.37 (1.88-2.99)***		2.74 (1.92-3.90)***		2.13 (1.56-2.91)***	
Proteinuria	1.55 (1.28-1.87)***		1.63 (1.22-2.16)***		1.49 (1.16-1.92)**	

Multivariate-adjusted^1^ age, duration of diabetes, smoking, alcohol drinking, exercising, hypertension, hypertension drug treatment, obesity, hyperlipidemia, type of DM treatment, and HbA_1_C. Multivariate-adjusted^2^ DKA, HHNK, severe hypoglycemia, stroke, myocardial infarction, peripheral neuropathy, intermittent claudication, and neuropathy in addition to variables in model 1.

*p<0.05; **p<0.01; ***p<0.001.

Table 3 shows the adjusted HR of all-cause mortality, mortality due to expanded and nonexpanded CVD-related causes for patients with an eGFR of 15–59.9 mL/min/1.73 m^2^ and 60–89 mL/min/1.73 m^2^ compared with those with an eGFR of 90 mL/min/1.73 m^2^or greater across the strata of proteinuria. Similarly, Table 4 shows the adjusted HRs of proteinuria for all-cause mortality and mortality due to expanded and non-expanded CVD-related causes were reported across strata of eGFR categories of 15–59, 60–89, and ≥90 mL/min/1.73 m^2^. We did not detect any significant interaction effects of eGFR and proteinuria on all-cause mortality and mortality due to expanded and non-expanded CVD-related causes.

**Table 3:** Hazard ratios (HRs) of all-cause mortality and mortality due to expanded and non-expanded CVD-related causes according to clinical stage of time-dependent annual mean eGFR stratified by proteinuria status (n=6,523)

	**HR (95% CI)**							
	**Without proteinuria (n=4,266)**				**Proteinuria (N=2,257)**			
**Variables**	**n**	**All-cause mortality**	**Expanded CVD-related mortality**	**Non-expanded CVD-related mortality**	**n**	**All-cause mortality**	**Expanded CVD-related mortality**	**Non-expanded CVD-related mortality**
Age-adjusted								
Annual mean eGFR (mL/min/1.73m^2^)								
≥90	2787	-	-	-	899	-		
60~89	1225	1.02 (0.75-1.39)	1.16 (0.72-1.86)	0.93 (0.62-1.40)	756	1.42 (1.03-1.96)*	1.62 (0.97-2.71)	1.31 (0.86-2.00)
15~59	254	2.22 (1.56-3.18)***	2.20 (1.26-3.83)**	2.28 (1.43-3.63)***	602	2.51 (1.86-3.39)***	3.36 (2.11-5.37)***	1.99 (1.34-2.96)***
P for trend		<0.001	0.01	0.009		<0.001	<0.001	<0.001
Multivariate-adjusted^1^								
Annual mean eGFR (mL/min/1.73m^2^)								
≥90	2787	-	-	-	899	-		
60~89	1225	1.13 (0.82-1.54)	1.33 (0.82-2.16)	1.00 (0.66-1.52)	756	1.56 (1.12-2.16)**	1.76 (1.05-2.95)*	1.45 (0.94-2.22)
15~59	254	2.46 (1.69-3.57)***	2.62 (1.47-4.70)**	2.39 (1.46-3.89)***	602	2.58 (1.90-3.51)***	3.28 (2.04-5.29)***	2.14 (1.42-3.22)***
P for trend		<0.001	0.003	0.005		<0.001	<0.001	<0.001
Multivariate-adjusted^2^								
Annual mean eGFR (mL/min/1.73m^2^)								
≥90	2787	-	-	-	899	-	-	-
60~89	1225	1.11 (0.81-1.52)	1.28 (0.79-2.08)	1.01 (0.67-1.53)	756	1.53 (1.10-2.12)*	1.70 (1.02-2.86)*	1.43 (0.94-2.20)
15~59	254	2.23 (1.51-3.30)***	1.98 (1.06-3.69)*	2.47 (1.49-4.10)***	602	2.49 (1.83-3.39)***	3.15 (1.96-5.09)***	2.06(1.37-3.12)***
P for trend		<0.001	0.04	0.006		<0.001	<0.001	<0.001

Multivariate-adjusted^1^ age, duration of diabetes, smoking, alcohol drinking, exercising, hypertension, hypertension drug treatment, obesity, hyperlipidemia and type of DM treatment and HbA_1_C. Multivariate-adjusted^2^ DKA, HHNK, severe hypoglycemia, stroke, myocardial infarction, peripheral neuropathy, intermittent claudication and neuropathy in addition to variables in model 1.

*:p<0.05; **:p<0.01; ***:p<0.001.

**Table 4:** Hazard ratios (HRs) of proteinuria for all-cause mortality and mortality due to expanded and non-expanded CVD-related causes stratified by eGFR status (n=6,523)

						**Baseline mean eGFR**	**HR (95% CI)**					
		**≥90 (N=3,686)**				**60~89 (N=1,981)**				**15~59 (N=856)**		
**Variables**	**n**	**All-cause mortality**	**Expanded CVD-related mortality**	**Non-expanded CVD-related mortality**	**n**	**All-cause mortality**	**Expanded CVD-related mortality**	**Non-expanded CVD-related mortality**	**n**	**All-cause mortality**	**Expanded CVD-related mortality**	**Non-expanded CVD-related mortality**
Age-adjusted												
Proteinuria												
No	2,787	1.00	1.00	1.00	1,225	1.00	1.00	1.00	254	1.00	1.00	1.00
Yes	899	1.60** (1.17-2.19)	2.10** (1.29-3.41)	1.33 (0.88-2.01)	756	2.35*** (1.79-3.10)	2.47*** (1.64-3.72)	2.26*** (1.56-3.29)	602	2.51*** (1.72-3.67)	2.70*** (1.55-4.70)	2.32** (1.37-3.93)
Multivariate-adjusted^1^												
Proteinuria												
No	2,787	1.00	1.00	1.00	1,225	1.00	1.00	1.00	254	1.00	1.00	1.00
Yes	899	1.34 (0.97-1.86)	1.78* (1.08-2.93)	1.11 (0.73-1.70)	756	2.09*** (1.58-2.77)	2.18*** (1.44-3.32)	2.03*** (1.38-2.97)	602	2.31*** (1.56-3.40)	2.39** (1.36-4.21)	2.17** (1.27-3.72)
Multivariate-adjusted^2^												
Proteinuria												
No	2,787	1.00	1.00	1.00	1,225	1.00	1.00	1.00	254	1.00	1.00	1.00
Yes	899	1.17 (0.83-1.66)	1.40 (0.80-3.46)	1.02 (0.65-1.60)	756	1.92*** (1.43-2.58)	1.96** (1.27-3.03)	1.93** (1.30-2.87)	602	2.37*** (1.60-3.52)	2.41** (1.36-4.28)	2.29** (1.33-3.95)

Multivariate-adjusted^1^ age, duration of diabetes, smoking, alcohol drinking, exercising, hypertension, hypertension drug treatment, obesity, hyperlipidemia and type of DM treatment and HbA_1_C. Multivariate-adjusted^2^ DKA, HHNK, severe hypoglycemia, stroke, myocardial infarction, peripheral neuropathy, intermittent claudication and neuropathy in addition to variables in model 1.

*:p<0.05; **:p<0.01; ***:p<0.001.

## Discussion

In this prospective study, a lower annual eGFR is predictive of all-cause as well as expanded and non-expanded CVD-related mortalities in patients with diabetes. The predictive ability of a lower annual eGFR values was based on the presence of proteinuria. Similarly, the predictive ability of proteinuria was based on the stages of renal function measured by eGFR. This finding is important because the current guidelines for the classification and staging of chronic kidney disease are based on eGFR, without considering concomitant proteinuria. Our findings provide the estimates of the risk stratification by eGFR along with proteinuria for Chinese patients with type 2 diabetes.

Our finding that lower GFR values are associated with a higher risk of all-cause mortality is consistent with previous studies [13,14,29-31]. In addition, studies have shown that lower eGFR values are associated with about a 3-fold higher risk of CVD mortality. Similar results were observed between the current study and those of Casale Monferrato Study and Nag et al., and the association in the Casale Monferrato Study did not reach statistical significance. In addition to more precise results, three major advantages were obtained using the methodology in the present study. One is that we measured time-varying mean annual eGFR. Second, previous studies did not consider co-morbidities and complications when they explored these associations. The possibility that co-morbidities and complications may explain the relationships observed was ruled out by further considering the effects of co-morbidities and complications. The associations still remained significant, although the effect was attenuated. Third, previous studies did not use both proteinuria and eGFR to predict mortality. This information would be potentially useful in informing ongoing discussions about how best to stratify the risk of mortality among patients with type 2 diabetes using both proteinuria and eGFR.

Several plausible explanations for the association between decreased renal function and mortality were identified. First, diabetic patients with decreased renal function have a higher prevalence of several CVD risk factors, including hypertension, dyslipidemia, and obesity, as well as complications. In our study, the prevalence of hypertension was 2.14 times higher, 1.29 times higher for dyslipidemia, 1.12 times higher for obesity, 3.55 times higher for stroke, 4.00 times higher for coronary artery disease, and 1.71 for myocardial infarction among patients with eGFR < 60 mL/min/1.73 m^2^ than among patients with eGFR ≥90 mL/min/1.73 m^2^. These chronic conditions resulted in decreased renal function. Decreased renal function itself may exert an independent effect on mortality. Another possible explanation is that decreased renal function results in lower clearance and higher plasma levels of inflammatory and oxidative stress factors, such as homocysteine and asymmetric dimethylarginine [37]. These unmeasured factors may explain the higher risk of mortality in patients with decreased renal function. In addition, decreased renal function may exacerbate hypertension and activate the rennin-angiotensin system [38], which may also increase the risk of mortality. These two biological mechanisms both aggravate atherosclerotic burden and potentially increase the risk of CVD.

Our findings have several clinical implications. First, decreased renal function is an important indicator of renal function in patients with diabetes. Proteinuria status, serum creatinine concentrations, and eGFR values of these patients should be routinely monitored. Second, lifestyle intervention and use of medications that can prevent the deterioration of renal function should be emphasized in diabetes care.

Our study has several strengths, including a large number of patients with diabetes, a long follow-up period, the use of a standardized procedure for data collection, and available information on a large number of potential confounding factors. In addition, we averaged repeated eGFR measurements to reduce random variation of measurement errors and improve precision. We also used time-varying eGFR to increase the accuracy of renal function measurement.

Our study has several limitations that need to be considered when interpreting our results. First, we did not screen other measures of impaired kidney function, such as microalbuminuria or cystatin C. Microalbuminuria may be a better marker of early kidney dysfunction, and cystatin C may be a better marker of chronic kidney disease than creatinine. Second, we used the MDRD equation to estimate GFR based on serum creatinine levels. Creatinine levels are affected by differences in muscle mass, and muscle mass distribution varies across gender and age. Although the MDRD equation was developed to consider population differences in muscle mass by age and gender, it does not consider individual differences [39]. Third, despite adjustments for a large number of potential confounders, including lifestyle behaviors, medications, and complications, residual and unrecognized confounding may be present because of the observational nature of our study. Lastly, all patients with type 2 diabetes in this study were enrolled in a Diabetes Care Management Program at a single medical center; thus, they may not be representative of all diabetic patients in Taiwan. In order to evaluate generalizability, we compare the age and gender distributions between the age and gender structure in our study and the national population with type 2 diabetes that was enrolled in the Nation Health Insurance program, which has a coverage rate of more than 99%. Similar distributions were found; the differences in proportions for categories of age and gender distributions between these two groups ranged from 0.56% to 5.24%. The non-differential distributions in age and sex indicate the representativeness of our study sample.

In conclusion, proteinuria and eGFR are independently predictive of all-cause and both expanded and non-expanded CVD-related mortalities in patients with type 2 diabetes, supporting the importance of renal function in mortality prevention. Furthermore, risk stratification by eGFR alone may be relatively insensitive to clinically relevant risk gradients. Therefore, the use of proteinuria measurement with eGFR increases the precision of risk stratification for mortality.

## Misc

Tsai-Chung Li and Sharon LR Kardia contributed equally to this work.

## Competing interests

The authors have no competing interests to declare.

## Authors’ contributions

C-CL and T-CL contributed equally to the design of the study and direction of its implementation, including supervision of the field activities, quality assurance and control. P-TK, C-CC, C-SL, W-YL and C-CL supervise the field activities. C-CL, S-LRK and T-CL helped conduct the literature review and prepare the Methods and the Discussion sections of the text. S-LRK, C-IL and S-YY designed the study’s analytic strategy and conducted the data analysis. All authors read and approved the final manuscript.
